# Gypenosides Alleviate Hyperglycemia by Regulating Gut Microbiota Metabolites and Intestinal Permeability

**DOI:** 10.3390/cimb47070515

**Published:** 2025-07-03

**Authors:** Rong Wang, Xue-Feng Liu, Kuan Yang, Li-Li Yu, Shao-Jing Liu, Na-Na Wang, Yun-Mei Chen, Ya-Qi Hu, Bei Qin

**Affiliations:** 1 Xi’an Key Laboratory for Research and Development of Innovative Multi-Target Anti-Hypertensive Drugs, Xi’an Medical University, Xi’an 710021, China; 2 Xi’an Innovative Anti-Hypertensive Drugs International Science and Technology Cooperation Base, Xi’an Medical University, Xi’an 710021, China; 3 Institute of Drug Research, Xi’an Medical University, Xi’an 710021, China; 4 College of Pharmacy, Xi’an Medical University, Xi’an 710021, China; 5 Shaanxi Institute of Food and Drug Control, Xi’an 710021, China

**Keywords:** gypenosides, type 2 diabetes mellitus, gut microbiota, short-chain fatty acids, bile acids

## Abstract

**Background/Objectives**: Gypenosides (Gps) are the main active compounds of *Gynostemma* and show promise in managing diabetes; nevertheless, the mechanism by which Gps exert anti-diabetic effects is still not fully understood. The aim of this study is to clarify the molecular mechanisms of Gps in ameliorating glucose dysregulation. **Methods**: Qualitative and quantitative analyses on the chemical components of Gps were performed, respectively. Type 2 diabetes mellitus mouse models were established, and the mice were subsequently treated with Gps at doses of 200, 100, or 50 mg/kg for 4 weeks. Biochemical markers were measured. Histopathological assessments of hepatic and colonic tissues were conducted. The compositions of the intestinal microbiota, short-chain fatty acids (SCFAs), and bile acids (BAs) in fecal samples were analyzed. Western blotting was applied to examine the activation of relevant signaling pathways. **Results**: Gps have potent regulatory effects on metabolic homeostasis by improving glucose and lipid profiles and alleviating hepatic tissue damage. Treatment with Gps significantly reduced serum levels of lipopolysaccharides and key pro-inflammatory cytokines (interleukin-6 and tumor necrosis factor-α). Moreover, Gps enhanced the integrity of the gut barrier by upregulating the level of tight junction proteins (ZO-1 and occludin). Microbiota profiling revealed that Gps markedly increased microbial diversity and richness, decreased the ratio of *Firmicutes*/*Bacteroidetes*, and elevated *Bacteroidia* abundance from the phylum to the genus level. Targeted metabolomics further demonstrated that Gps modulated gut microbial metabolites by promoting SCFA production and reshaping BA profiles. Specifically, Gps elevated the primary-to-secondary BA ratio while reducing the 12α-hydroxylated to non-12α-hydroxylated BA ratio. Mechanistically, Western blotting demonstrated that Gps triggered the hepatic PI3K/AKT pathway and the intestinal BA/FXR/FGF15 axis, suggesting the coordinated regulation of metabolic and gut–liver axis signaling pathways. **Conclusions**: Gps significantly ameliorate hyperglycemia and hyperlipidemia through a multifaceted mechanism involving gut microbiota modulation, the restoration of intestinal barrier function, and the regulation of microbial metabolites such as SCFAs and BAs. These findings offer novel insights into their mechanism of action via the gut–liver axis.

## 1. Introduction

Type 2 diabetes mellitus (T2DM) is a metabolic disorder marked by persistent hyperglycemia [1]. The global prevalence of T2DM continues to rise sharply. Currently, more than 350 million individuals are affected by T2DM worldwide, and this figure could rise to reach approximately 439 million by the year 2030 [2]. Although various antidiabetic drugs are widely used in clinical practice, many are associated with adverse effects, including cardiovascular complications [3], gastrointestinal disturbances [4], weight gain, and acute pancreatitis [5]. Given these limitations, the exploration of natural products as potential therapeutic agents is of considerable importance in the search for safer and more effective options for managing T2DM.

*Herba Gynostemmatis*, commonly known as “JiaoGuLan” in Chinese, refers to the whole herb or aerial parts of several species within the genus *Gynostemma* (*Cucurbitaceae* family). Widely distributed throughout Asia, *Gynostemma* species are well recognized for their dual use as food and traditional medicine [6,7]. As a traditional Chinese medicinal herb, *Gynostemma* has been historically used for addressing various ailments, including liver disorders [8], tumors [9], diabetes [10], and hyperlipidemia. The presence of gypenosides (Gps), flavonoids, and polysaccharides in plants of the *Gynostemma* genus has been confirmed through phytochemical analysis [11]. Gps have been reported to be major bioactive compounds with a series of dammarane-type triterpene tetracyclic saponins with a similar structure to that of ginsenosides [12]. Prior research studies have demonstrated that Gps possess anti-hyperglycemia effects through enhancing the Nrf2 signaling pathway [13], reducing oxidative stress, and interacting with the AGE-RAGE signaling cascade [14].

The intestinal microbiota ecosystem plays a vital role in maintaining metabolic homeostasis and regulating various physiological functions [15]. Gut microbiota is intricately involved in the pathophysiological processes associated with the onset and progression of T2DM. The dysbiosis of the gut microbiota can disrupt the integrity of the intestinal barrier, leading to increased gut permeability, the activation of inflammatory responses, and ultimately facilitating the progression of insulin resistance and T2DM [16]. Microorganisms and their metabolic byproducts, particularly microbial-derived metabolites, are now recognized as key modulators of host metabolic pathways, including glucose regulation.

Short-chain fatty acids (SCFAs), which are the main end-products of the microbial fermentation of dietary fibers, function as critical signaling molecules that mediate communication between the intestinal microbiota and the organism [17]. SCFAs influence T2DM progression by promoting insulin secretion, enhancing insulin sensitivity, and modulating gluconeogenesis within the gut. For example, acetic acid and butyric acid reinforce gut barrier integrity, improve glycogen metabolism, and attenuate inflammation, thereby contributing to glycemic control in diabetic individuals [18,19]. Bile acids (BAs) represent another important class of microbiota-derived metabolites with essential roles in cholesterol homeostasis and metabolic regulation. Alterations in BA metabolism—often driven by microbial imbalances—have been implicated in the development of hyperglycemia and hyperlipidemia [20]. In patients with T2DM, both the composition and the proportion of BAs are frequently disrupted. Modifying the BA pool and restoring its balance have emerged as a potential strategy for improving glucose metabolism and insulin sensitivity [21]. Thus, targeting the intestinal microbiota and their metabolites, such as SCFAs and BAs, represents a potential therapeutic approach for the management of T2DM.

Although the existing literature provides evidence supporting the hypoglycemic effects of Gps, the pharmacological role of Gps in modulating glucose metabolism remains insufficiently characterized. In the current research study, we systematically investigated the effects of Gps on glucose metabolism using a mouse model of T2DM, which was developed through the combination of a high-fat diet (HFD) and streptozotocin (STZ) treatment. Additionally, we explored the interactions among gut microbiota, SCFAs, and BAs, as well as the involvement of relevant signaling pathways, to elucidate the mechanisms underlying the glucose modulating properties of Gps.

## 2. Materials and Methods

### 2.1. Chemical Compounds and Reagents

The medicinal materials (*Gynostemma longipes* C.Y. Wu—as confirmed on http://www.cn-flora.ac.cn, accessed on 7 October 2024) were sourced from the *Gynostemma* pentaphyllum planting base, which has attained national GAP certification. The standard substances of Gypenoside A (Gps A) (No. 112093-202201, 97.8%) and Gypenoside XLIX (Gps XLIX) (No. 112033-201902, 97.2%) were bought from the National Institutes for Food and Drug Control. High-fat diet (Dossy Experimental Animal Co., Ltd., Chengdu, China) formula was composed of basic diet (70%), sucrose (16%), lard (12%), cholesterol (1%), and sodium cholate (1%). Streptozotocin (STZ, CAS No. 18883-66-4) and metformin (H20023370) were supplied by Med Chem Express (Monmouth Junction, NJ, USA) and Merck (Nanjing, China), respectively. Assay kits for total cholesterol (TC), triglycerides (TG), low-density lipoprotein cholesterol (LDL-C), and high-density lipoprotein cholesterol (HDL-C) were acquired from Nanjing Jiancheng Bioengineering Institute (Nanjing, China). Additionally, enzyme-linked immunosorbent assay kits (ELISA) for interleukin-6 (IL-6), insulin, lipopolysaccharide (LPS), and tumor necrosis factor-α (TNF-α) were acquired from Shanghai Yuanju Biotechnology Co., Ltd. (Shanghai, China). The primary antibodies for PI3K p85 (Proteintech, Wuhan, China), Akt (CST, Danvers, MA, USA), p-Akt (CST), FGF15 (Abcam, Cambridge, UK), FXR/NR1H4 (HUABIO, Hangzhou, China), Occludin (Abcam), zonula occludens-1 (ZO-1) (Servicebio, Wuhan, China), and GAPDH (Proteintech) were used. All reagents and chemical compounds in this research were of analytical grade.

### 2.2. Extract Preparation and Qualitative and Quantitative Analyses of Gps

Briefly, dried *Gynostemma longipes* was powdered to pass through a 20-mesh sieve and extracted three times with 70% ethanol at 75 °C for 60 min each time. The extract samples were combined and concentrated under reduced pressure to remove the ethanol. Then, the crude extract was obtained. Subsequently, the crude extract was purified using an AB-8 macroporous adsorption resin column. Initially, the column was washed with water to elute the water-soluble impurities. After that, it was eluted with 70% ethanol. Finally, the eluates were collected, concentrated, and freeze-dried to obtain the Gps extract powder [22].

A qualitative assessment of Gps based on ultra-performance liquid chromatography–electrospray ionization–quadrupole time-of-flight tandem mass spectrometry (UPLC-ESI-Q/TOF-MS/MS) method was established. Separation was accomplished using a Waters BEH C_18_ column (2.1 × 100 mm, 1.8 μm) with column oven set at 30 °C. The eluent was composed of phase A (0.1% formic acid) and phase B (acetonitrile). The gradient elution was performed according to the following schedule: 5% B was held constant from 0 to 1 min, then gradually increased from 5% to 50% over 1 to 13 min; the percentage of B was increased gradually from 50% to 100% from 13 to 30 min, followed by a decrease from 100% to 5% from 30 to 32 min; and, finally, 5% B was maintained for an additional 2 min. Utilizing an injection volume of 5 μL and a flow rate of 0.2 mL/min, an electrospray ionization source was operated in dual-ionization mode to obtain MS analysis within a mass range of *m*/*z* from 50 to 1000. For MS detection, the detection parameters were set as follows: the capillary voltage was set to 2.0 kV, the desolvation temperature was maintained at 500 °C, the gas flow for desolvation was adjusted to 800 L/h, the cone gas flow was maintained at 50 L/h, and the temperature of the source was regulated at 120 °C.

Quantitative analysis of the chemical marker content in Gps was performed using high-performance liquid chromatography (HPLC) on the Shimadzu LC-30AD system (Shimadzu, Kyoto, Japan). The system was fitted with a diode array detector (DAD) and paired with the Thermo Hypersil Gold C18 column, measuring 4.6 × 250 mm with a particle size of 5 μm. Acetonitrile (A) and water (B) with the following gradient profile: 0–15 min, 25–40% A; 15–25 min, 40–30% A; 25–30 min, 30–25% A; and 30–40 min, 25–25% A. The temperature of the column was maintained at 30 °C throughout the analysis. The detection wavelength was set to 203 nm. The flow rate of the mobile phase was maintained at 1.0 mL/min, and the volume of the sample injected into the system was 10 μL.

### 2.3. Animal Models and Treatments

Male C57BL/6 mice were used (weighing approximately 18 ± 2 g; Dossy Experimental Animal Co., Ltd., Chengdu, China). All animal protocols were approved under the approval number SCXK (Chuan) 2020-0030. All mice were kept in a monitored environment where the temperature was controlled at 23 ± 3 °C and a relative humidity of 50–60% was maintained under a 12 h light/12 h dark cycle. They were offered water and food ad libitum. After adaptive feeding phase, the experimental mice were randomly assigned to two groups: one group was the normal control (NC) group (*n* = 6), while the other group was the model group (*n* = 30). The NC mice were fed a standard diet, whereas the model mice were given HFD. Following a period of 4 weeks, the model group received 120 mg/kg STZ through a single intraperitoneal injection [23]. The NC group received an identical volume of 0.1 M citrate buffer. Seven days after the single STZ injection, bloods were obtained via the tail vein, the fasting blood glucose (FBG) level was measured using a glucometer (Sinocare Inc., Changsha, China), and FBG > 11.1 mmol/L over three consecutive measurements was considered indicative of T2DM status having been successfully attained. T2DM mice were further randomized into the following five groups with six animals each: a diabetes model control (MC) group, a metformin group at 150 mg/kg (Met), low-dose Gps group at 50 mg/kg (Gps-L), moderate-dose Gps group at 100 mg/kg (Gps-M), and high-dose Gps group at 200 mg/kg (Gps-H). Throughout the experiment, the Met and Gps groups were administered metformin or Gps daily via gavage. The NC and MC groups were treated with saline every day.

Weekly monitoring was conducted for body weight (BW) and FBG levels. The food and water intake of each mouse was determined. After 4 weeks of intervention and 12 h fasting duration, an oral glucose tolerance test (OGTT) was conducted. Mice received a gavage of 2.0 g glucose/kg, and blood glucose levels were determined at 0, 30, 60, 120, and 180 min [23]. Subsequently, blood was withdrawn via retro-orbital puncture and the serum was separated for biochemical assays. Mice were sacrificed via cervical dislocation, and the liver and colon tissue samples were collected. Fresh feces were promptly collected and then stored at −80 °C for the subsequent gut microbiota, SCFA, and BA analyses.

### 2.4. Blood Biochemical Tests

The blood lipid levels were quantified using commercially available kits. The plasma insulin, LPS, IL-6, and TNF-α levels in each mouse were assayed by using ELISA kits. Additionally, the assessment of insulin resistance through the homeostasis model (HOMA-IR) was computed based on the following formula:

HOMA-IR = FBG × insulin/22.5

### 2.5. Histological Staining

The hepatic tissues were promptly fixed with 4% paraformaldehyde, subjected to dehydration, and subsequently embedded for hematoxylin and eosin (H&E) staining. In addition, oil red O and periodic acid-schiff (PAS) staining were performed on hepatic tissues. Microscopic imaging of the tissue sections was subsequently performed using an upright light microscope (3DHISTECH Pannoramic 250, Budapest, Hungary).

### 2.6. Immunohistochemistry of Colon Tissues

Colon tissues were promptly set in 4% paraformaldehyde for further analysis and then embedded for sectioning. Colonic slices were incubated with anti-mouse Occludin and ZO-1 antibodies at 4 °C overnight. Following this incubation period, horseradish peroxidase-tagged secondary antibodies were introduced to the sections to facilitate the detection of the primary antibodies. The tissue sections were subsequently stained using 3,3′-Diaminobenzidine (ZSGB-BIO, Beijing, China) along with hematoxylin. The BA200 digital microscope (Mike Audi Industrial Group Co., Ltd., Xiamen, China) was employed to capture microscopic images. Quantitative analysis was then performed with Halo 101-WL-HALO-1 software (Indica Labs, Albuquerque, NM, USA).

### 2.7. 16S rDNA Sequencing Analysis

Fecal sample DNA was extracted, as per a previously reported method [24]. Polymerase chain reaction (PCR) testing was employed to amplify the V3–V4 segment of bacterial 16S rRNA genes, and the resulting DNA fragments were observed using 2% agarose gel electrophoresis. The concentration of the amplified DNA was quantified by using a fluorescence-based quantification method. Subsequently, equal amounts of the amplified DNA were pooled together and subjected to paired-end 2 × 250 bp sequencing on the Illlumina MiSeq platform fitted with MiSeq Reagent Kit v3 (Illumina, San Diego, CA, USA). To comprehensively evaluate the diversity within the gut microbiota, diverse analyses were performed, including α/β diversity analysis, differential species abundance analysis, identification of biomarker species, and functional prediction.

### 2.8. SCFA Analysis

The samples were combined with 500 μL of distilled water and homogenized using 100 mg of glass beads for 1 min. Following this, the mixture was centrifuged at 12,000 rpm for 10 min. Next, 200 µL of the resulting supernatant was transferred into a new microtube, where 100 µL of 15% phosphoric acid was added for the extraction process. A 20 μL internal standard (375 μg/mL 4-methylvaleric acid) was incorporated into acidified fecal homogenate along with 280 μL of ether. The mixture underwent a homogenization process that lasted for 1 min and then was recentrifuged at 12,000 rpm and 4 °C for 10 min. After centrifugation, the supernatant was carefully collected to facilitate the subsequent analysis of SCFAs employing gas chromatography–MS (GC-MS).

### 2.9. BA Analysis

The determination of BAs was conducted as described in previous study [25]. Fecal samples of 20 mg were extracted with 495 μL methanol. Then, 5 μL of the internal standard solution was incorporated into each sample. The solutions were held at −20 °C for 10 min. Subsequently, the samples underwent centrifugation at 12,000 rpm and 4 °C for another 10 min. After the centrifugation process, the supernatant was transferred and filtered through a protein precipitation plate in preparation for UPLC–tandem MS (UPLC-MS/MS) analysis.

### 2.10. Western Blotting

The protein was isolated from hepatic tissues using RIPA buffer (Pioneer Biotechnology, Xi’an, China). An equivalent amount of protein was subjected to 10% SDS polyacrylamide gel electrophoresis, followed by transferal onto PVDF membranes. The membranes underwent blocking with 5% nonfat milk for 2 h at room temperature and were subsequently probed with different primary antibodies overnight at 4 °C. Next, membranes were treated with appropriate HRP-conjugated secondary antibodies for 2 h. Visualization of the bands was performed using enhanced chemiluminescence (Proteintech Biotechnology) using real-time quantitative Western blot detection and analysis system (LuQAS1000, Xi’an, China, ShaanXi Pioneer), and the analysis was conducted with Image J software (version 1.37V). The relative expressions of target proteins were normalized against GAPDH as a reference.

### 2.11. Statistical Analysis

Data were displayed as mean ± standard error of the mean (SEM). Statistical analyses were performed utilizing SPSS 10.0. All data were subjected to normality testing using the Shapiro–Wilk test and equal variance testing using Brown–Forsythe test.

For comparisons among the experimental groups, one-way analysis of variance (ANOVA) along with Tukey’s multiple comparison test were employed for statistical comparisons between the experimental groups. Spearman’s correlation analysis was used to determine the correlation coefficients between gut microbiota and various indicators. Statistical significance was set at *p* < 0.05.

## 3. Results

### 3.1. Chemical Components of Gps Measured by UPLC-ESI-Q/TOF-MS/MS

**Figure 1:**
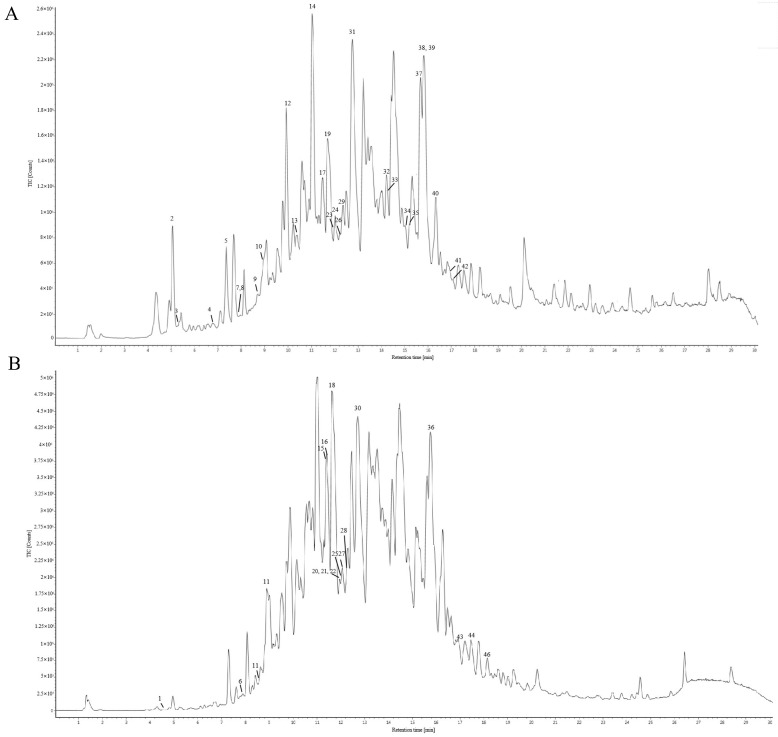
Total ion chromatography of the chemical composition of gypenosides in positive ion mode (**A**) and negative ion mode (**B**).

The chemical constituents of Gps extracted from *Gynostemma longipes* were analyzed using UPLC-ESI-Q/TOF-MS/MS. The total ion chromatogram is presented in Figure 1. Based on fragmentation patterns in both positive and negative ion modes, the retention times, and database comparisons, 46 chemical compounds were tentatively identified. Of these, 33 were classified as saponins. Comprehensive details of these components are provided in Supplementary Material Table S1.

### 3.2. Quantitative Analysis of Key Chemical Markers

**Figure 2:**
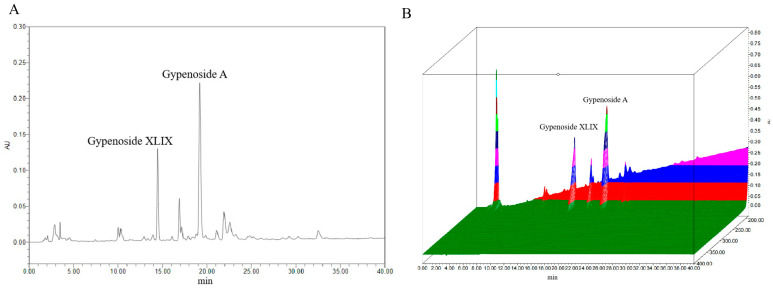
Typical chromatogram (**A**) and 3D chromatogram (**B**) of marker components.

Previous studies have identified Gp XLIX and Gp A as two predominant neutral Gps commonly used as chemical markers; particularly, Gp XLIX is a characteristic component of *Gynostemma longipes* used for quality evaluations and the standardization of extracts of Gps. The contents of Gp XLIX and Gp A were detected according to the national pharmaceutical standard (WS3-Z-006-93(Z)). A representative chromatogram is presented in Figure 2. Quantitative analyses were performed in duplicate, and the average levels of the analytes were calculated and are expressed in mg/g (Table 1). Specifically, the levels of Gp XLIX and Gp A were determined to be 5.34–5.45 mg/g and 10.14–10.36 mg/g, respectively.

### 3.3. Gps Exhibit Metabolic Protective Effects in HFD/STZ-Induced Diabetic Mice

**Figure 3:**
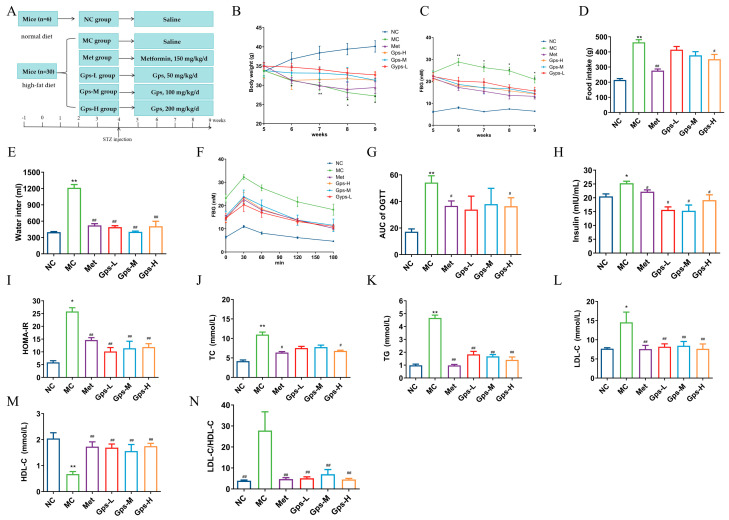
Effects of Gps on physiological metabolism. Experimental protocol and schedule (**A**), Body weight (**B**), Fasting blood glucose (FBG) (**C**), Food intake (**D**), Water intake (**E**), Time course of blood glucose levels (**F**) and AUC during oral glucose tolerance test (**G**), Insulin (**H**) and homeostasis model assessment of insulin resistance (HOMA-IR) (**I**), Total cholesterol (TC) (**J**), Triglycerides (TGs) (**K**), Low-density lipoprotein cholesterol (LDL-C) (**L**), High-density lipoprotein cholesterol (HDL-C) (**M**), and LDL-C/HDL-C (**N**) in mice. The data are expressed as mean ± SEM (*n* = 6). * *p* < 0.05 and ** *p* < 0.01 denote significance relative to the NC group; ^#^ *p* < 0.05 and ^##^ *p* < 0.01 denote significance relative to the MC group.

The experimental design and timeline are illustrated in Figure 3A. Compared with the NC group, mice in the MC group exhibited typical symptoms of diabetes, including increased food and water intake, weight loss, and elevated FBG levels. The oral administration of Met and Gps for 4 weeks significantly ameliorated these symptoms to varying extents (Figure 3B–E). While the MC group displayed a gradual decline in hyperglycemia, both the Met- and Gps-treated groups exhibited a rapid reduction in blood glucose levels, approaching baseline values. The OGTT results and the calculated area under the curve (AUC) showed improved glucose tolerance in the Gps-H and Met groups (*p* < 0.05) (Figure 3F,G). Moreover, the MC group demonstrated considerably higher levels of serum insulin and HOMA-IR when compared to the NC group (*p* < 0.05). Following treatment with either Met or Gps, insulin and HOMA-IR values were markedly reduced (*p* < 0.05 or *p* < 0.01), suggesting insulin sensitivity was improved (Figure 3H,I). Collectively, these findings demonstrate that Gps significantly enhances glucose tolerance and reduces insulin resistance in diabetic mice.

Dysregulated lipid metabolism often accompanies hyperglycemia in T2DM. In the MC group, the TG, TC, and LDL-C levels were greatly elevated, whereas HDL-C levels were notably decreased compared with the NC group. Treatment with Gps-H or Met resulted in substantial reductions in the concentrations of TG, TC, and LDL-C (Figure 3J–L), while HDL-C levels were markedly increased (*p* < 0.01) (Figure 3M). To further evaluate lipid metabolic disorders, the LDL-C/HDL-C ratio—a known indicator of cardiovascular risk—was calculated. This ratio was significantly reduced in the Gps group (4.71 ± 0.67) compared with the MC group (27.80 ± 0.67) (*p* < 0.01) (Figure 3N), indicating improved lipid profiles. Overall, these findings suggest that Gps effectively alleviate dyslipidemia in T2DM mice.

### 3.4. Gps Alleviate Liver Histology of HFD/STZ Mice 

**Figure 4:**
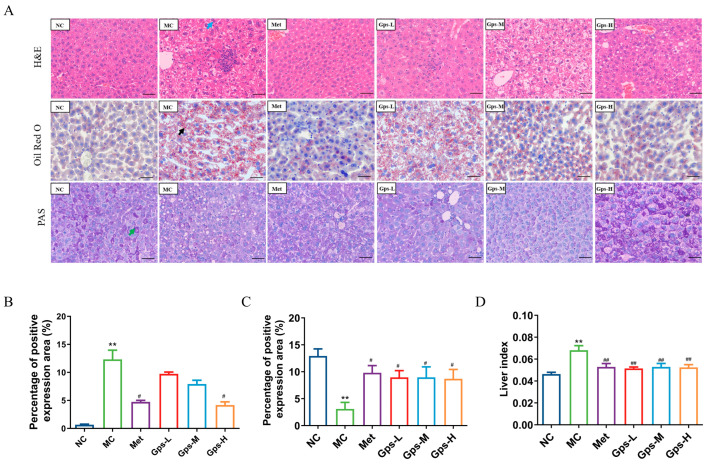
Effects of Gps on liver histopathology. Typical hematoxylin–eosin (H&E), oil red O, and periodic acid-schiff (PAS) staining of liver tissue (**A**) (scale bar: 50 μm). Lipid accumulation score (**B**), PAS staining corresponding score (**C**), Liver index of the mice (**D**). The the blue arrow represented hepatocellular steatosis, the black arrow represented lipid droplets. The data are expressed as mean ± SEM (*n* = 6). ** *p* < 0.01 denote significance relative to the NC group; ^#^ *p* < 0.05 and ^##^ *p* < 0.01 denote significance relative to the MC group.

The liver index of the mice significantly decreased following treatment with Gps (Figure 4D, *p* < 0.05). A histological examination revealed that liver tissues from the NC group exhibited a normal architecture without the infiltration of inflammatory cells. By contrast, the MC group displayed marked hepatocellular steatosis and the infiltration of inflammatory cells. The intervention with Gps notably mitigated these pathological changes. Both Met and Gps treatments significantly reduced hepatic lipid droplet accumulation. Moreover, the lipid-droplet-positive area in liver tissue was markedly lower in the Gps-H group (4.18 ± 0.41%) than the MC group (12.32 ± 1.18%) (Figure 4B, *p* < 0.05).

Liver glycogen, a key reservoir for maintaining blood glucose levels, was evaluated using PAS staining to assess glycogen metabolism in T2DM. As shown in Figure 4C, glycogen deposition was significantly reduced in the MC group (3.09 ± 0.88%) compared with the NC group (12.92 ± 0.95%) (*p* < 0.01). Following Gps treatment, glycogen storage was substantially restored (*p* < 0.01), indicating an improvement in hepatic glucose metabolism.

### 3.5. Gps Modulate Gut Microbiota Composition in T2DM Mice

**Figure 5:**
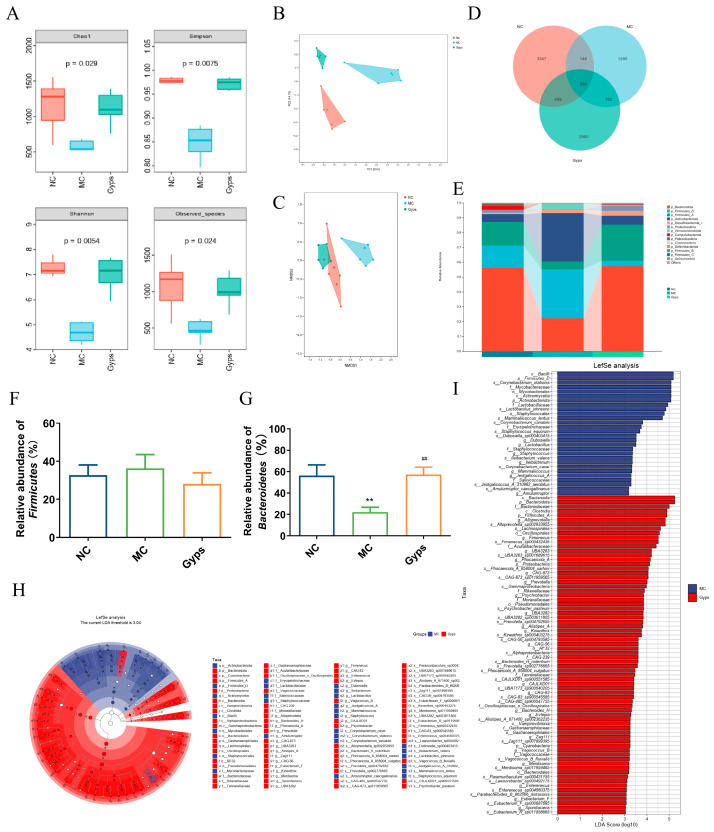
Impacts of Gps on the diversity and relative abundance of gut microbiota. The ɑ diversity of gut microbiota (**A**), Principal coordinates (PCoA) with Bray–Curtis distances on operational taxonomic unit (OTU) (**B**), Non-metric multidimensional scaling (NMDS) at OTU level with Bray–Curtis distances (**C**), Venn diagram based on all OTU data (**D**), Relative abundance of microbial communities at phylum level (**E**), Relative abundance of *Firmicutes* (**F**) and *Bacteroidetes* (**G**), and Taxonomic cladogram (**H**) and histogram (**I**) based on LEfSe analysis. LDA scores > 3.0. *p* < 0.05 showed significant differences. ** *p* < 0.01 denote significance relative to the NC group; ^##^ *p* < 0.01 denote significance relative to the MC group.

To further explore the impact of Gps on gut microbiota, 16S rRNA sequencing was performed on 18 samples from the NC, MC, and Gps-H groups (referred to as the Gps group hereafter). An alpha diversity analysis—using Chao1, Simpson, Shannon, and observed species indices—revealed that the Gps treatment significantly increased microbial richness and diversity (Figure 5A). A beta diversity assessment was conducted utilizing principal coordinate analysis (PCoA) and non-metric multidimensional scaling, which were based on Bray–Curtis distances calculated at the operational taxonomic unit (OTU) level (Figure 5B,C). The microbial communities clustered distinctly by groups, suggesting significant differences in gut microbiota composition.

A Venn diagram illustrated that there were 590 OTUs common to all groups, while unique OTUs numbered 3347 in the NC group, 1295 in the MC group, and 2960 in the Gps group (Figure 5D). At the phylum level, the gut microbiota primarily comprised *Bacteroidota*, *Firmicutes*, *Actinobacteriota*, *Desulfobacterota*, *Proteobacteria*, and *Verrucomicrobiota* (Figure 5E). Compared with the NC group, the MC group exhibited a marked reduction in *Bacteroidota* abundance. Notably, Gps administration significantly elevated *Bacteroidota* abundance, increasing from 22.13 ± 4.69% in the MC group to 57.25 ± 6.99% in the Gps group (*p* < 0.01) (Figure 5G), thereby reducing the *Firmicutes*/*Bacteroidetes* (F/B) ratio. Alterations in the intestinal microbiota have been linked to T2DM pathogenesis, particularly through metabolite-mediated effects on insulin resistance and energy metabolism. An elevated F/B ratio is commonly associated with T2DM [23]. Thus, the observed decrease in the F/B ratio following Gps treatment may contribute to its protective effects on glucose and lipid metabolism. Linear discriminant analysis effect size (LEfSe) was employed to recognize bacteria that were differentially enriched between the MC and Gps groups (Figure 5H,I). The analysis revealed 28 taxa augmented in the MC group and 72 taxa enriched in the Gps group. The Gps treatment significantly increased the abundance of several genera, including *Alloprevotella*, *Fimenecus*, *Phocaeicola_A*, *Prevotella*, *Psychrobacter*, *Alistipes_A*, *Kineothrix*, *Bacteroides_H*, *Evtepia*, *Vagococcus_B*, *Merdisoma*, *Enterenecus*, *Eubacterium_F*, *Sporofaciens*, and various unclassified or uncultured bacteria. Notably, Gps had a pronounced impact on the *Bacteroides* genus, significantly enhancing its abundance at both the genus and phylum levels. These findings suggest that the modulation of the gut microbiota underlies the metabolic improvements observed with the Gps treatment.

### 3.6. Gps Inhibit Inflammation and Alleviate Gut Barrier Damage

**Figure 6:**
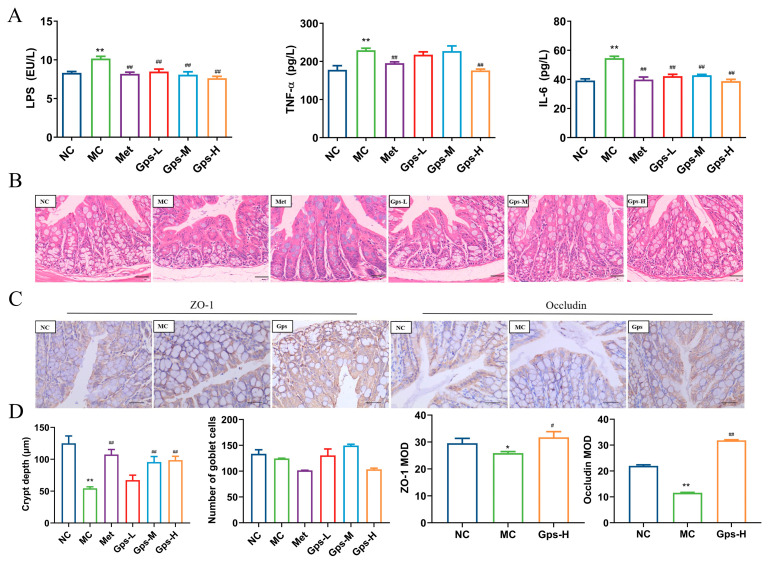
Effects of Gps on inflammation and intestinal integrity. The content of LPS and inflammation markers (**A**); HE staining of colon tissue (scale bar: 20 μm) (**B**); Immunohistochemical staining of ZO-1 protein and occludin protein (scale bar: 40 μm) (**C**); The crypt depth, number of goblet cells, and mean optical density (MOD) of ZO-1 and occludin analyzed with Image-Pro Plus (**D**). The data are expressed as mean ± SEM (*n* = 6). * *p* < 0.05 and ** *p* < 0.01 denote significance relative to the NC group; ^#^ *p* < 0.05 and ^##^ *p* < 0.01 denote significance relative to the MC group.

LPS, the outer membrane component of Gram-negative bacteria, has the ability to move from the gut into systemic circulation, where it triggers the release of inflammatory mediators and cytokines, thereby exacerbating gut barrier damage [26]. In the MC group, the levels of LPS, TNF-α, and IL-6 were significantly elevated, indicating a pronounced inflammatory response. The treatment with Gps markedly reduced the levels of these inflammatory markers (Figure 6A). Histological analysis of the colon showed that the MC group demonstrated a marked decrease in the depth of the cecal crypts, indicative of mucosal atrophy. The treatment with Gps reversed these histological abnormalities and significantly increased the depth of the colonic crypts (Figure 6B). Furthermore, the protein expressions of tight junction markers ZO-1 and occludin were markedly reduced in the MC group relative to the NC group. The administration of Gps notably enhanced the expression levels of both ZO-1 and occludin (*p* < 0.01 or *p* < 0.05; Figure 6C,D), suggesting its protective effect on gut barrier integrity.

### 3.7. Gps Increase SCFA Production in T2DM Mice

**Figure 7:**
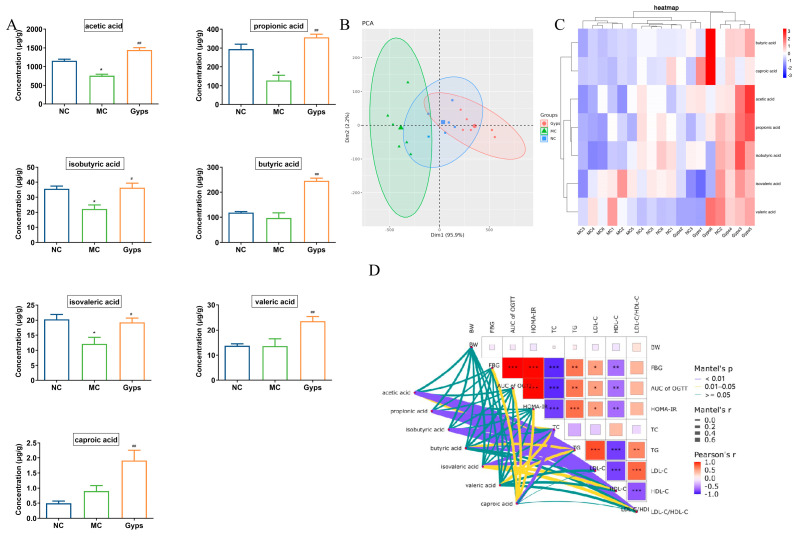
Effects of Gps on short-chain fatty acid (SCFAs) content in feces. Histogram of the 7 SCFAs (**A**), Principal component analysis (PCA) plot of SCFAs in each group (**B**), Heatmap of the contents of different groups of SCFAs (**C**), The correlation between glucose- and lipid-metabolism-related indexes and SCFAs using Mantel’s analysis (**D**). The data are expressed as mean ± SEM (*n* = 6). * *p* < 0.05 and ** *p* < 0.01 denote significance relative to the NC group; ^#^ *p* < 0.05 and ^##^ *p* < 0.01 denote significance relative to the MC group. * denote the significance of correlation in subfigures (**D**), * *p* < 0.05, ** *p* < 0.01 and *** *p* < 0.001.

Given the above results demonstrating that Gps modulate gut microbiota composition, we next examined their impact on SCFAs, which are produced by the microbial fermentation of undigested carbohydrates and have well-established health benefits [27]. A quantitative analysis of fecal SCFA levels revealed that concentrations of acetic acid, propionic acid, isobutyric acid, and isovaleric acid were markedly decreased in the MC group compared with the NC group. The administration of Gps significantly reversed these declines (*p* < 0.05 or *p* < 0.01; Figure 7A). Notably, butyric acid, valeric acid, and caproic acid levels were significantly increased following the Gps treatment compared with the MC group (*p* < 0.01). A principal component analysis (PCA) further supported these findings, showing distinct clustering of samples across groups, indicative of substantial differences in SCFA profiles (Figure 7B). The heatmap clustering of the SCFA concentrations revealed that the profile of the Gps group closely resembled that of the NC group (Figure 7C). These results suggest that SCFA production, markedly reduced in T2DM mice, was restored by the Gps treatment. Moreover, Mantel’s analysis demonstrated significant correlations between SCFA levels and multiple glucose- and lipid-metabolism-related parameters, including FBG, OGTT AUC, HOMA-IR, and particularly lipid indices such as TC, TG, LDL-C, HDL-C, and the LDL-C/HDL-C ratio (*p* < 0.01 or *p* < 0.05; Figure 7D).

### 3.8. Gps Improve BA Dysregulation in T2DM Mice

**Figure 8:**
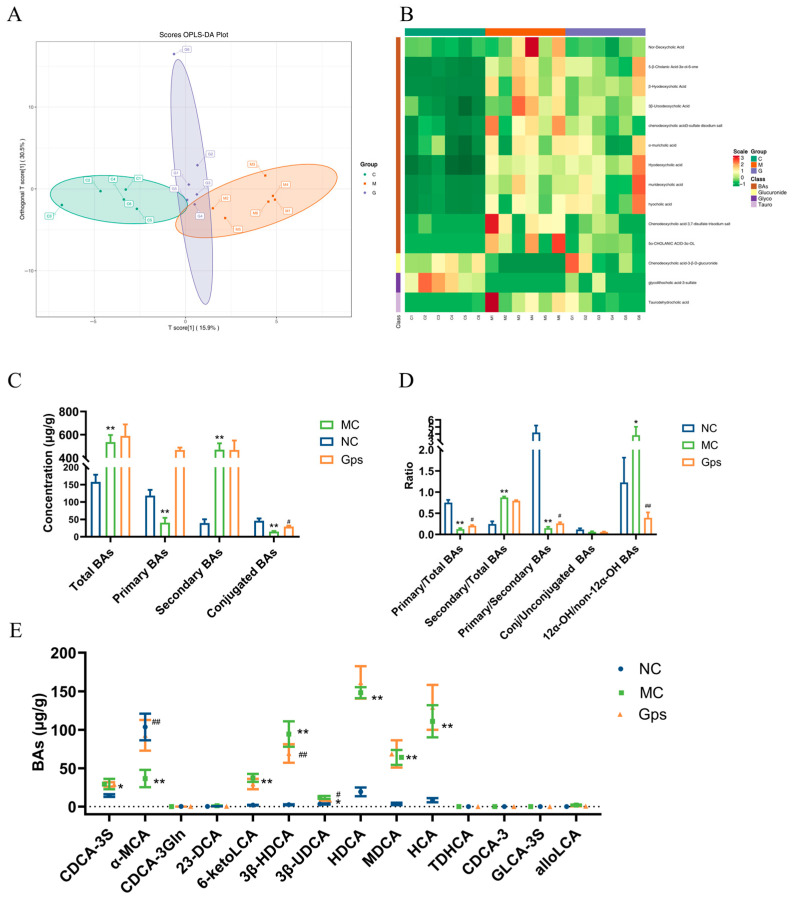
Effects of Gps on bile acid (BA) distribution and composition in feces. The discriminant analysis of orthogonal partial least squares (OPLS-DA) score plots for different groups (**A**); Heatmap of 14 BA components (**B**); The concentrations of total BAs, primary BAs, secondary BAs, and conjugated BAs (**C**); the ratio of primary/total BAs, secondary/total BAs, primary/secondary BAs, conj/unconjugated BAs and 12α-OH/non-12α-OH BAs (**D**); The content of BAs regulated by Gps (**E**). The data are expressed as mean ± SEM (*n* = 6). * *p* < 0.05 and ** *p* < 0.01 denote significance relative to the NC group; ^#^ *p* < 0.05 and ^##^ *p* < 0.01 denote significance relative to the MC group.

An orthogonal partial least squares discriminant analysis revealed clear separation between the NC and MC groups, indicating marked differences in BA profiles in T2DM mice (Figure 8A). Notably, the Gps group clustered closer to the NC group, suggesting that the Gps treatment ameliorated BA disturbances. There were variable levels of importance in the projection analysis, which identified 14 significantly altered BAs, including 23-DCA, 6-ketoLCA, 3β-HDCA, 3β-UDCA, and CDCA-3S. These are presented in the hierarchical clustering heatmap in Figure 8B. The MC group displayed elevated levels of total and secondary BAs and reduced levels of primary and conjugated BAs compared with the NC group (Figure 8C). Moreover, the ratios of secondary/total BAs and 12α-OH/non-12α-OH BAs were significantly elevated in the MC group (0.87 ± 0.058 and 3.84 ± 1.21), whereas the Gps treatment decreased these ratios (0.79 ± 0.039 and 0.39 ± 0.13). Conversely, the ratios of primary/total BAs and primary/secondary BAs, which were reduced in the MC group (0.13 ± 0.058 and 0.15 ± 0.078) following the HFD/STZ, were significantly increased by the Gps treatment (0.21 ± 0.039 and 0.26 ± 0.065) (Figure 8D). The levels of the 14 BAs across groups are shown in Figure 8E. Collectively, these findings suggest that Gps effectively ameliorate BA dysregulation in T2DM mice.

### 3.9. Effects of Gps on Regulating Signal Pathways Involved in Insulin and BA Metabolism

**Figure 9:**
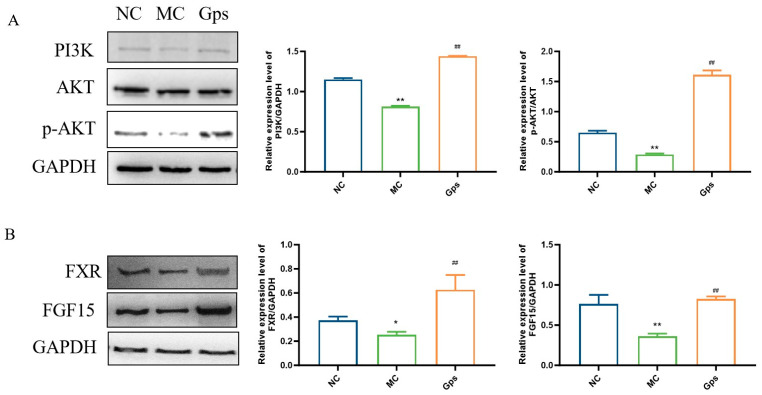
Effects of Gps on the regulation of the protein expressions involved in insulin and BA metabolism. Representative Western blot bands and quantitative analysis of the expression of proteins associated with the PI3K/Akt pathway (**A**) and FXR/FGF15 pathway (**B**). The data are expressed as mean ± SEM (*n* = 6). * *p* < 0.05 and ** *p* < 0.01 denote significance relative to the NC group; ^##^ *p* < 0.01 denote significance relative to the MC group.

The PI3K/Akt signaling pathway serves as a crucial downstream mediator of insulin and plays a pivotal role in the regulation of glucose and lipid metabolism [28]. Additionally, the intestinal FXR/FGF15 axis is a primary regulatory pathway for BA biosynthesis within enterohepatic circulation [29]. To investigate the potential mechanisms that explain the effects of Gps, we conducted an analysis of protein expression levels related to the PI3K/Akt and FXR/FGF15 signaling pathways using Western blotting. In the MC group, the expression of PI3K and the ratio of phosphorylated Akt (p-Akt) to total Akt were significantly reduced, indicating impaired insulin signaling. In contrast, the Gps treatment markedly increased the expressions of PI3K and the p-Akt/Akt ratio (MC group: 0.81 ± 0.015 and 0.29 ± 0.024; Gps group: 1.44 ± 0.0067 and 1.61 ± 0.12, *p* < 0.01) (Figure 9A), suggesting the restoration of insulin pathway activity. Similarly, the expressions of intestinal FXR and FGF15 were significantly lower in the MC group compared with the NC group. The administration of Gps reversed these reductions, significantly upregulating FXR and FGF15 protein expression (MC group: 0.25 ± 0.040 and 0.36 ± 0.058; Gps group: 0.63 ± 0.21 and 0.83 ± 0.052, *p* < 0.01) (Figure 9B). These results suggest that Gps modulate both insulin and BA metabolism by activating the hepatic PI3K/Akt pathway as well as the intestinal FXR/FGF15 axis.

### 3.10. Correlation Analysis Between Gut Microbial Communities and Metabolic Parameters (SCFAs and BAs)

**Figure 10:**
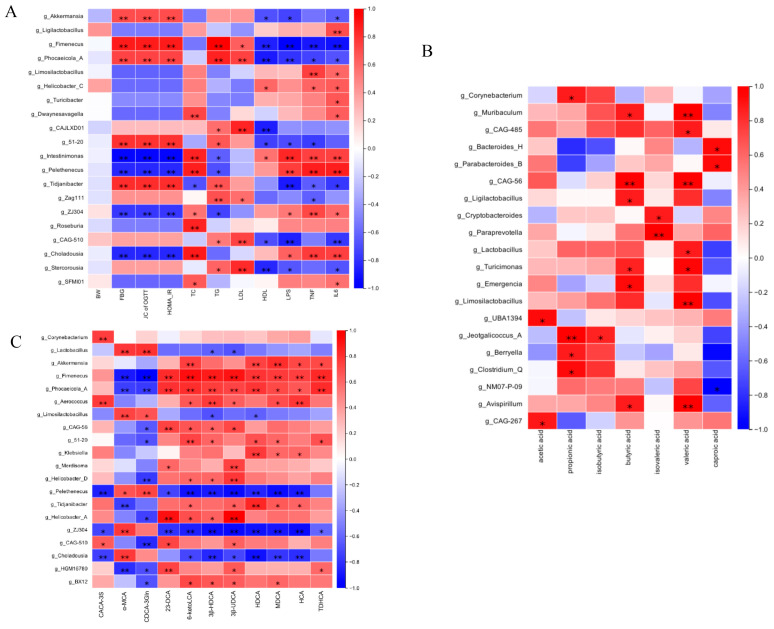
Spearman’s analysis of correlation between gut microbiota and glucose- and lipid-metabolism-related indexes (**A**), SCFAs (**B**), and BAs (**C**). * denote the significance of correlation, * *p* < 0.05 and ** *p* < 0.01.

To explore the functional relationships between intestinal microbiota compositional shifts and host metabolic changes, Spearman’s correlation test was conducted, resulting in a comprehensive correlation matrix. This analysis examined associations among gut microbial genera, glucose- and lipid-metabolism-related parameters, and pro-inflammatory cytokines (Figure 10A). At the genus level, *Akkermansia*, *Fimenecus*, and *Phocaeicola* were positively correlated with glucose and lipid metabolism indicators and inversely associated with pro-inflammatory cytokines. *Intestinimonas* and *Pelethenecus* exhibited an inverse correlation. Figure 10B further demonstrated that variations in gut microbial abundance directly influenced SCFA levels. Additionally, Figure 10C illustrates the relationships between microbial genera and fecal BA composition. Specifically, the relative abundance of *Akkermansia* and *Fimenecus* was positively associated with levels of secondary BAs such as 6-ketoLCA, HDCA, MDCA, HCA, and TDHCA. Conversely, primary BAs including α-MCA and CDCA-3Gln were negatively associated with *Lactobacillus*. These findings indicate that Gps-induced modifications in gut microbiota composition are closely linked to SCFA and BA profiles, further corroborating the role of microbiota–metabolite interactions in metabolic regulation.

## 4. Discussion

Diabetes mellitus is the most prevalent chronic metabolic disorder, characterized by hyperglycemia along with disturbances in lipid, carbohydrate, and protein metabolism. Natural products have long been recognized as abundant sources of antidiabetic agents, contributing significantly to both clinical practice and experimental research in the treatment of diabetes. Among these natural compounds, saponins—such as ginsenosides and notoginsenosides—are considered major bioactive constituents due to their potent hypoglycemic effects. In this research, Gps were isolated from *Gynostemma longipe* located in the Qinba mountain region. We assessed the quality of Gps by examining chemical markers (Gp XLIX and Gp A) [30]. The findings from the experiments indicated that the content of Gps complies with national pharmaceutical standards.

In the present study, a mouse model of T2DM was created using a combination of an HFD and STZ to evaluate the effects of an intervention of Gps on glucose- and lipid-metabolism-related parameters. The administration of Gps significantly reduced both hyperglycemia and hyperlipidemia in diabetic mice. Insulin resistance is one of the hallmark pathophysiological features of diabetes, and glucose intolerance plays a pivotal role in the progression of T2DM. Our results demonstrated that the levels of FBG, insulin, HOMA-IR, and the AUC for OGTTs after 4 weeks of Gps treatment were significantly different when compared to those of the MC group and were reduced by 25.36–32.06%, 24.25–39.44%, 54.08–60.70%, and 30.01–37.52%, respectively. Moreover, the Gps treatment induced a dose-dependent decrease in TC, TG, and LDL-C levels, while simultaneously elevating HDL-C levels. These results indicated that Gps, which significantly enhance glucose tolerance and reduce insulin resistance, exert therapeutic benefits on both hyperglycemia and hyperlipidemia in T2DM mice. Consequently, we further explored the potential mechanisms underlying the effects of Gps.

T2DM is strongly linked to chronic inflammation and impaired gut barrier function. LPS, a key structural component of the outer membrane of Gram-negative bacteria, can translocate from the intestinal lumen into systemic circulation, thereby increasing plasma levels of pro-inflammatory cytokines such as TNF-α and IL-6 [31]. A high-fat, high-cholesterol diet not only contributes to the onset of T2DM but also exacerbates gut dysbiosis and compromises intestinal barrier integrity. This dysfunction facilitates the translocation of LPS into the bloodstream, thereby promoting low-grade chronic inflammation, a well-established factor in the pathogenesis and progression of T2DM [32]. Proteins from tight junctions—including claudins, ZO-1, and occludin—are critical for preserving intestinal barrier function and regulating gut permeability [33]. Our study demonstrated that Gps treatment significantly decreased serum concentrations of LPS, TNF-α, and IL-6 while upregulating the ZO-1 and occludin expression. A histological examination of colon tissue also revealed improved structural integrity following the administration of Gps. These findings suggest that Gps can mitigate LPS-induced gut barrier dysfunction by enhancing intestinal integrity and reducing systemic inflammation.

Accumulating evidence suggests that intestinal microbiota and their metabolites are essential for pathogenesis of T2DM [34]. Among these, microbiota-derived metabolites—particularly SCFAs and BAs—are key mediators of host–microbe interactions [35]. In mice, the gut microbiota is predominantly made up of three key bacterial phyla: *Proteobacteria*, *Bacteroidetes*, and *Firmicutes*. Characteristic changes in intestinal microbial composition in T2DM are often observed, marked by a rise in *Firmicutes* and a reduction in the proportion of *Bacteroidota*, resulting in a higher F/B ratio—a microbial signature frequently associated with metabolic disorders [23]. Consistent with previous studies, the results showed that treatment with Gps led to a reduction in the F/B ratio, decreasing from 2.20 ± 0.73 in the MC group to 0.59 ± 0.18 in the Gps group, and a significant increase in *Bacteroidia* abundance across multiple taxonomic levels, from phylum to genus. Notably, genera such as *Prevotella*, *Alloprevotella*, *Bacteroides_H*, *Phocaeicola_A*, and *Alistipes_A*—all members of the *Bacteroidetes* phylum—were enriched following the intervention with Gps. *Prevotella* contributes to maintaining an acidic gut microenvironment and suppressing inflammatory responses, while *Alloprevotella* is among the most prevalent beneficial bacteria known to be involved in the regulation of blood glucose through interactions with natural compounds [36]. These results indicated that the antidiabetic effects of Gps may be at least partly mediated by the modulation of gut microbiota composition in T2DM mice.

To further elucidate the mechanisms by which gut microbiota may regulate glucose and lipid metabolism through Gps, we performed targeted metabolomic profiling to examine microbial metabolites, specifically SCFAs and BAs. *Bacteroides* are particularly important for fermenting complex carbohydrates into SCFAs [35], and a lower F/B ratio enhances SCFA production by altering intestinal microbiota [37]. In agreement with previous observations, our results indicated that the administration of Gps improved SCFA levels in diabetic mice. Correlation analysis using Spearman’s method revealed significant positive associations between SCFA levels and the abundance of several bacterial genera, including *Corynebacterium*, *Muribaculum*, *Bacteroides*, *Lactobacillus*, *Clostridium*, and *Parabacteroides*. Many of these genera—particularly *Bacteroides*, *Lactobacillus*, and *Clostridium*—are known SCFA producers and are associated with anti-inflammatory effects [38,39]. These results indicate that Gps not only enhances the population of SCFA-producing microbiota but also increases SCFA production, thereby contributing to the mitigation of inflammation and improvement of metabolic homeostasis in T2DM.

In addition to SCFAs, emerging evidence highlights the role of BAs as critical agents in the crosstalk between intestinal microbiota and host metabolism, influencing glucose tolerance, insulin sensitivity, and energy homeostasis [40]. These findings suggest that BAs represent a viable therapeutic target for the management of T2DM [40]. In the current study, the total BA levels in the MC group were notably higher in comparison to the NC group. Remarkably, the administration of Gps further increased BA levels in a sustained and progressive manner. The onset of T2DM is associated with hepatic lipid accumulation, which accelerates the conversion of excess cholesterol into BAs and promotes their excretion, thereby elevating fecal BA levels. The Gps treatment appeared to enhance this hepatic transport and excretory process, leading to further increases in BA output. Furthermore, an analysis of fecal samples revealed a reduction in the ratio of primary to secondary BAs and an increase in the ratio of 12α-hydroxylated to non-12α-hydroxylated BAs within the MC group, consistent with prior studies on insulin resistance and diabetic models [41,42]. Gut microbiota facilitate the transformation of primary BAs into secondary BAs through enzymatic pathways such as deconjugation, dehydroxylation, oxidation, and isomerization. The correlation analysis in this study indicated a positive association between secondary BAs and the relative abundance of *Akkermansia* and *Faecalibacterium*, while primary BAs were negatively correlated with *Lactobacillus* and *Faecalibacterium*. *Akkermansia* is known for its mucin-degrading capacity and choline production, whereas *Lactobacillus* contributes to SCFA and BA biosynthesis [43]. The observed modulation of the gut microbiota by Gps, particularly the decrease in the 12α-OH/non-12α-OH BA ratio and increase in the primary/secondary BA ratio, suggests a shift toward a more metabolically favorable BA profile.

The nuclear receptor FXR, activated by BAs, is a central regulator of BA homeostasis, controlling both synthesis and enterohepatic circulation [44]. The activation of intestinal FXR upregulates FGF15, which exerts downstream metabolic effects. Western blot analyses confirmed the increased protein expression of FXR and FGF15 in response to Gps treatment, indicating enhanced FXR pathway activity. These alterations may stimulate the FXR/FGF15 signaling axis, contributing to the attenuation of metabolic dysfunction associated with T2DM.

## 5. Conclusions

Overall, this study demonstrated that Gps exhibit significant potential in modulating glucose and lipid metabolism. Gps effectively alleviated hyperglycemia, hyperlipidemia, and systemic inflammation, ameliorated hepatic pathological damage, and preserved intestinal barrier integrity. The underlying mechanisms involve the reinforcement of the intestinal barrier, regulation of the intestinal microbiota composition, modulation of the synthesis of SCFAs, and alteration of BA profiles, thereby activating the intestinal BA/FXR/FGF15 axis and the hepatic PI3K/AKT signaling pathway. Collectively, these findings offer new perspectives to the multifaceted mechanisms by which Gps modulate metabolic functions and highlight their promise as a therapeutic candidate for the prevention and management of T2DM. However, additional studies were conducted to further verify the association between the gut microbiota and the anti-hyperglycemic effect of GPs, with the aim of revealing the potential glucose-modulating mechanisms of GPs.

## Figures and Tables

**Table 1:** Content of phytochemical markers in Gps for the validation of the HPLC method.

	Compound	Content (%)	Regression Equation	Linear Range (mg/mL)	Correlation Coefficient, r
1	Gp XLIX	5.34–5.45	*Y* = 64,757X − 13,585	0.02876–15	0.9999
2	Gp A	10.14–10.36	*Y* = 86,806X + 9016.5	0.02876–15	0.9999

## Data Availability

Data are contained within the article or Supplementary Material.

## Supplementary Materials

The following supporting information can be downloaded at: https://www.mdpi.com/article/10.3390/cimb47070515/s1.

## Abbreviations

The following abbreviations are used in this manuscript:

T2DM	Type 2 diabetes mellitus
Gps	Gypenosides
BW	Body weight
STZ	Streptozotocin
HFD	High-fat diet
FBG	Fasting blood glucose
OGTT	Oral glucose tolerance test
HOMA-IR	Homeostasis model assessment of insulin resistance
TC	Total cholesterol
TG	Total triglyceride
LDL-C	Low-density lipoprotein cholesterol
HDL-C	High-density lipoprotein cholesterol
TNF-α	Tumor necrosis factor-α
IL-6	Interleukin-6
LPS	Lipopolysaccharide
PCoA	Principal coordinates analysis
NMDS	Non-metric multidimensional scaling
OTU	Operational taxonomic unit
PCA	Principal component analysis
ZO-1	Zonula occludens-1
MOD	Mean optical density
SCFAs	Short-chain fatty acid
BAs	Bile acids
FXR	Farnesoid X receptor
