# Supplementary material for: Gypenosides Alleviate Hyperglycemia by Regulating Gut Microbiota Metabolites and Intestinal Permeability
Source: Curr Issues Mol Biol. 2025 Jul 3;47(7):515. doi: 10.3390/cimb47070515 (PMC12293292; doi:10.3390/cimb47070515)
Supplement: Supplementary file 1 [file cimb-47-00515-s001.zip › cimb-3703974-supplementary.pdf]

Table S1 Predicted chemical components of G. *longipes* identified *via* UPLC-Q-TOF-MS^E analysis

No.	RT (min)	Selected ion	MS (m/z)	Fragmentation (m/z)	Mass error (ppm)	Formula	Component name
1	4.63	$[M-H]^-$	153.0223	135.04484	9.4	$C_7H_6O_4$	Pyrocatechuic acid
2	5.07	$[M+Na]^+$	147.0432	147.04322	2.8	$C_7H_8O_2$	Guasol
3	5.16	$[M+K]^+$	432.2147	150.09169	-1.4	$C_{42}H_{74}O_{14}$	Gypenoside XXX
4	6.8	$[M+NH_4]^+$	169.0758	147.04324	4.5	$C_{16}H_{14}O_6$	Homoeriodictyol
5	7.38	$[M+H]^+$	465.1028	204.10116, 303.04875	0	$C_{21}H_{20}O_{12}$	Isoquercitrin
6	7.91	$[M-H]^-$	623.163	315.05047, 593.15132, 285.03953	2	$C_{28}H_{32}O_{16}$	Isorhamnetin-3-O-nehesperidine
7	7.96	$[M+H]^+$	317.0648	147.04371	-2.3	$C_{16}H_{12}O_7$	Isorhamnetin
8	7.97	$[M+H]^+$	625.1776	147.04371, 317.06492	2.1	$C_{36}H_{60}O_8$	Ginsenoside Rh(4)
9	8.92	$[M+Na]^+$	177.1275	177.12746	14	$C_{10}H_{18}O$	(-)-isomenthone
10	9.24	$[M+Na]^+$	1117.5661	107.04971, 471.34694, 921.48685, 574.20364	0.1	$C_{53}H_{90}O_{23}$	Gypenoside LVI
11	9.30	$[M-H]^-$	1045.5681	913.51821, 751.46932, 605.41032, 473.36424	1.5	$C_{52}H_{86}O_{21}$	Gypenoside XLIX
12	9.93	$[M+K]^+$	321.2218	313.25215	8.6	$C_{18}H_{34}O_2$	Oleic acid
13	10.66	$[M+H]^+$	303.0498	297.25760, 199.14934, 145.10114	-0.5	$C_{15}H_{10}O_7$	Quercetin
14	11.08	$[M+H]^+$	949.5400	313.25233, 619.42259	3.5	$C_{47}H_{80}O_{19}$	Gypenoside XLIV
15	11.39	$[M-H]^-$	1091.5982	1061.59309, 621.43399, 899.50822	-2.3	$C_{54}H_{92}O_{22}$	Gypenoside XXXV
16	11.41	$[M-H]^-$	1061.5947	179.05660, 783.48948, 899.50822	4.2	$C_{53}H_{90}O_{24}$	Gypenoside LXI
17	11.53	$[M+Na]^+$	969.5442	105.06918, 201.16389, 313.25266, 421.34681, 439.35737	5	$C_{48}H_{82}O_{18}$	Gypenoside XVII
18	11.64	$[M-H]^-$	553.2936	459.38334, 161.04573	-1.1	$C_{54}H_{92}O_{23}$	Ginsenoside Rb1
19	11.67	$[M+Na]^+$	807.4895	407.36621, 229.19525, 425.37717	3.7	$C_{42}H_{72}O_{13}$	Gypenoside LXXV
20	11.74	$[M-H]^-$	538.7899	131.03730, 489.35710	-5.5	$C_{53}H_{90}O_{22}$	Gypenoside LXIII
21	11.74	$[M-H]^-$	1093.5665	131.03730, 915.53366, 1031.58194	2	$C_{53}H_{90}O_{23}$	Gypenoside LXX
22	11.75	$[M-H]^-$	929.5458	398.23205, 131.03730, 915.53366	-2.3	$C_{48}H_{82}O_{17}$	Gypenoside LXXIII
23	11.82	$[M+H]^+$	423.3617	297.25769, 145.10096	4.7	$C_{28}H_{48}O$	Campesterol

No.	RT (min)	Selected ion	MS (m/z)	Fragmentation (m/z)	Mass error (ppm)	Formula	Component name
24	12.02	$[M+H]^+$	287.0551	191.17954	0.3	$C_{15}H_{10}O_6$	Kaempferol
25	12.06	$[M-H]^-$	961.5402	915.53115, 651.37605, 315.05046, 885.52140	2.5	$C_{48}H_{82}O_{19}$	Gypenoside XLVI
26	12.17	$[M+H]^+$	1109.6017	421.34589, 299.27337, 791.45574, 939.52750	2.17	$C_{54}H_{92}O_{23}$	Gypenoside XLII
27	12.25	$[M-H]^-$	915.5360	131.03557, 765.44360	4.1	$C_{47}H_{80}O_{17}$	Gypenoside IX
28	12.32	$[M-H]^-$	961.5409	131.03557, 403.28479, 535.32660, 765.44360	3.3	$C_{48}H_{82}O_{18}$	Ginsenoside Rd
29	12.39	$[M+H]^+$	753.4815	201.16364, 435.32560, 453.33663	4.2	$C_{41}H_{68}O_{12}$	Ginsenoside Rg6
30	12.88	$[M-H]^-$	931.5304	913.51661, 885.52114, 825.50085, 329.23320	3.4	$C_{47}H_{80}O_{18}$	Gypenoside LVII
31	13.14	$[M+Na]^+$	763.4737	339.23174, 283.16908, 469.33083	3.6	$C_{40}H_{68}O_{12}$	Gypenoside LXXVII
32	14.31	$[M+Na]^+$	953.5089	313.25272, 791.46359	0.9	$C_{47}H_{78}O_{17}$	Gypenoside LII
33	14.35	$[M+Na]^+$	661.4310	313.25272,643.38278	3.7	$C_{36}H_{62}O_9$	Gypenoside LXXVI
34	15.09	$[M+K]^+$	512.2415	437.34060	6.3	$C_{48}H_{82}O_{18}$	Ginsenoside Re
35	15.2	$[M+Na]^+$	777.4797	191.17988, 439.35717	4.8	$C_{41}H_{70}O_{12}$	Gypenoside XIII
36	15.7	$[M-H]^-$	783.4903	737.48774	0.4	$C_{42}H_{72}O_{13}$	Ginsenoside F2
37	15.76	$[M+Na]^+$	661.4319	440.36373, 237.07628	5	$C_{36}H_{62}O_9$	Gynosaponin TN-1
38	15.83	$[M+Na]^+$	659.4176	189.16414, 437.34072, 295.24205, 565.38899	7.1	$C_{36}H_{60}O_9$	Ginsenoside Rh8
39	15.83	$[M+Na]^+$	823.4841	191.17937, 437.34072, 565.38899	3.3	$C_{42}H_{72}O_{14}$	Gypenoside L
40	15.98	$[M+Na]^+$	805.4736	439.35630, 659.41519	3.4	$C_{42}H_{70}O_{13}$	Damulin A
41	16.85	$[M+H]^+$	540.3062	184.07339, 437.34077	5.1	$C_{53}H_{90}O_{22}$	Ginsenoside Rb2
42	17.02	$[M+H]^+$	919.5246	889.48908, 435.32622, 516.37576	-1.6	$C_{47}H_{80}O_{18}$	Gypenoside LXIV
43	17.17	$[M-H]^-$	929.5524	277.21737, 703.44374	4.8	$C_{48}H_{82}O_{17}$	Gypenoside XI
44	17.42	$[M-H]^-$	797.5088	751.46651, 505.35082	3.9	$C_{42}H_{72}O_{14}$	Gypenoside LXXIV
45	17.55	$[M+Na]^+$	805.4754	657.39714, 453.33628	5.7	$C_{42}H_{70}O_{13}$	Damulin B
46	18.21	$[M-H]^-$	897.4927	765.4478, 681.3884, 535.3289, 403.2844	8	$C_{46}H_{74}O_{17}$	Gypenoside A
